# Equity and Social Care in Medicaid through Community Care Hubs

**DOI:** 10.1017/amj.2025.10089

**Published:** 2025-12

**Authors:** John V. Jacobi

**Affiliations:** 1 Seton Hall Law School, Newark, United States; 2 Yale Law School, New Haven, United States

**Keywords:** Medicaid, Value Based Payment, Health Related Social Needs, Health Equity, Medicalization, Care Coordination, Community Care Hubs

## Abstract

Medicaid serves vulnerable populations that experience deep health inequities and significant health-related social needs. In recent years, reforms to Medicaid have sought to respond to those needs, with mixed results. Value based payment methods, which in theory link payment to outcome metrics, are emerging in commercial insurance markets and can be adapted to the needs of Medicaid programs and their beneficiaries. These methods seek to tie payment for services to the forging of connections between medical and social care including housing supports and nutrition services for vulnerable populations. This paper describes the merits and some pitfalls of the attempt to turn Medicaid from its roots as a medical insurance program to a broader health insurance program. It describes the benefits of employing community care hubs – intermediaries between community-based organizations and large payers and hospital systems – as a way to spur the move to social care in Medicaid. It also addresses some of the barriers to this move, including the perceived danger of “medicalizing” society’s failures and the apparent turn by the current federal administration away from commitment to health equity.

Medicaid serves vulnerable populations that experience deep health inequities and significant health-related social needs.^1^ Medicaid has over the decades attempted to design its payment system to respond to those needs.^2^ Recent analyses establish the troubling persistence of these inequities and needs, and cast about for methods for Medicaid to address them.^3^ The use of value-based payments (“VBPs”) to support community intermediaries is a way forward.

This paper will describe the inequities and social care needs that impede beneficiaries from using their Medicaid benefits to improve their health status. Previous efforts to reform Medicaid to improve the health status of the most vulnerable have fallen short of the mark. Community Care Hubs (“CC Hubs”) have the capacity address the equity shortfall in current Medicaid. CC Hubs have existed for many years on a small scale and have had some success in connecting Medicaid payments to providers in a way that addresses some of the root causes of health inequity and social service deficits.^4^

This is a systems problem. The theory of VBPs, through which payment for health services depends not on the volume of services provided but on the value of those services as defined by the payer can improve the functionality of Medicaid system.^5^ A reconfiguration of the relationship between the payer and provider as mediated by CC Hubs has some promise to fill a gap that has frustrated reformers of Medicaid for decades.

In any health care finance and delivery system there is a payment to provider process. Funds for care are raised through taxes, insurance premiums, employer contributions, and out of pocket payments. The funds are then directed to pay for the cost of care provided to the beneficiaries of public or private systems of care. The mechanics of this translation from raising funds to laying on of hands is complex and varies from nation to nations and within the United States from payer to payer.^6^ The way we pay for care matters. Effective throughput of funding to achieve equitable, appropriate care to those in need of services depends on many factors, including choosing appropriate participating caregivers,^7^ designing an effective means of identifying those services subject to reimbursement,^8^ and less formal influences such as the moral compass of caregivers reflected in professional ethics codes.^9^

Part I of this paper will provide an overview of efforts to use VBPs to drive reimbursement toward payers’ economic and social goals. VBPs, like other means of addressing the payment to provision process, condition payment on provision of care consistent with payer preferences and leading to preferred outcomes.^10^ This part will describe the means by which VBPs are intended to contain cost, improve equity in the health system, and expand wellness by encouraging attention to health-related social needs (“HRSNs”).^11^ It also addresses concerns that VBPs are simply new wine in old bottles, especially those employed in the Medicaid setting. To the extent VBPs structure the payment process as a series of incentives to drive beneficial outcomes, they may recapitulate the mixed results of previous similarly motived efforts such as Medicaid managed care. Medicaid managed care programs were designed to contain cost but also to improve quality of care and address equity concerns with scant success.^12^ Can VBP-driven Medicaid programs do better? This Part introduces CC Hubs as a mechanism capable of preventing frustration of the promise of VBPs, so long as their use is accompanied by appropriately sensitive regulatory oversight.

Part II more fully describes CC Hubs.^13^ CC Hubs sit as intermediaries between upstream, large actors and providers of care. They contract with the moneyed actors such as managed care organizations, large hospital systems, and large self-funded health plans.^14^ But they also contract with downstream, small actors, often community-based organizations (“CBOs”) focused on one or more aspects of social care responsive to patients’ HRSN, including housing supports, nutrition counseling, and employment counseling.^15^ CC Hubs’ value lies in their ability to connect these two sets of entities. In VBP contractual settings featuring whole person care, the large actors have incentives to produce “value” extending to addressing patients’ HRSN, but little contact with community resources able to tackle those needs.^16^ CBOs, on the other hand, have the capacity and the mission to address the identified social needs, but are not engaged in the payment system as are, for example, Medicaid providers.^17^ CC Hubs can form relationships with CBOs and upstream providers to communicate service needs, success (or failures) to be documented, and steer funding to support the social services provided.

This part then describes the regulatory measures needed to ensure that VBPs in Medicaid, and specifically the use of CC Hubs, avoid the pitfalls that have bedeviled some previous attempts to drive payment systems to appropriate care. Attention to cost containment cannot be permitted to eclipse the goals of equity and whole person care, permitting the focus on HRSNs to fall by the wayside. The CC Hubs should be mission driven. Their limited role of providing funding and technical support to CBOs and connecting those CBOs to upstream actors can prevent the dilution of their attention and a drift to simple cost control.

Part III briefly addresses two sources of tension with the vision articulated in Part II. First, it addresses the “medicalization” criticism of many forms of whole person care.^18^ The medicalization debate centers on whether engaging the health care delivery system and/or Medicaid to remediate social problems experienced by patients could have the detrimental effects of pathologizing conditions deriving from failed social systems and depriving advocates of improved housing, employment, and nutritional services of leadership roles that they have earned over years of hard work.^19^ This part argues that these concerns can be exaggerated, and to the extent they point to real dangers, the CC Hub model’s focus on supporting CBOs ameliorates those dangers. Second, whole person care and the CC Hubs are threatened by regulatory shifts in the second Trump administration. While Medicaid support has not yet been withdrawn, guidance provided by the Centers for Medicaid and CHIP Services has raised concerns.^20^ These regulatory indications are concerning, but with some funding adjustments the pilots advancing them may receive continued funding.

## I. Can VBPs succeed where other methods have failed to drive equity and access in Medicaid?

A central problem in the U.S. health system is that high levels of spending on health care has not resulted in similarly high levels of population health status as compared to other nations’ experience.^21^ How should our system change? Much of the scholarship on the health finance and delivery system in recent years has focused on front-end funding questions, often framed as a variant of “Medicare for All”: who pays for coverage, who gets covered, how a reformed national health system might be structured,^22^ and what the normative principles of such reform should be.^23^ Other have approached reform from the other end of the telescope: how should physician practices change their operations to improve the outcomes of care delivery.^24^

This paper deals with a subset of this systemic problem in two senses: first, it addresses Medicaid as an (important) corner of the system. Second, it addresses not the “front end” (who pays for care) or the “back end” (how do physicians organize their practices), but rather the connection between these two nodes: how do payers transmit signals to providers as conditions of payment to encourage the results they seek?^25^ I have previously referred to this as the “last mile” problem.^26^ Payers are interested in cost control. But payers are increasingly focused on access to social care, in recognition that HRSNs are significant drivers of health access and equity gains for vulnerable populations including those served by Medicaid.^27^ Successful systems responses to these problems require payment methods that signal a need to address health equity and HRSNs.

### A. Medicaid managed care has not solved the equity problem

Medicaid managed care was intended to provide those signals but has had mixed results. A study-of-studies published in 2012 and funded by the Robert Wood Johnson Foundation reported that studies of Medicaid managed care found mixed evidence regarding increased access to care,^28^ and little evidence of improved quality of care.^29^ There may be a lag in the reporting of studies of Medicaid managed care. A 2023 publication of the Medicaid and CHIP Payment and Access Commission (“MACPAC”) found that even by that time “[i]t [was] not clear whether managed care provides better or worse access to care than” fee for service Medicaid, and that “[f]indings on Medicaid managed care quality outcomes are scarce and have mixed results.”^30^

These studies of Medicaid managed care disclose that tools intended to improve program access, quality, and cost have not significantly advanced program quality and access.^31^ Can VBP methods do any better at least in terms of improving health equity and access to social care necessary to improve health status? Under VBP models, providers’ payments are conditioned on “their performance on select quality and efficiency measures” through financial incentives.^32^

VBPs “aim to drive system change towards greater efficiency and improved health outcomes. In contrast to traditional fee-for-service payment models that are based on the volume of care provided, value-based payment models reward providers based on achievement of quality goals and, in some cases, cost savings.”^33^ Sylvia Burwell signaled a comprehensive turn to VBPs in 2015 when she announced that the Centers for Medicare and Medicaid Services was intent to “find better ways as a country to deliver care, pay providers, and distribute information” by “moving from volume to value in Medicare payments.”^34^ Secretary Burwell expressed the belief that this movement to value was in line with innovations in state Medicaid programs, health insurers, and self-funded plans.^35^

### B. Properly regulated VBPs can improve equity in Medicaid

Medicaid managed care showed little benefit in terms of cost savings, patient access, or quality of care.^36^ One reason for the small changes accomplished by shifting Medicaid to managed care is that managed care plans “have only a limited ability to change traditional delivery systems and an equally limited ability to respond to the social determinants of health that play a large role in the fragmented care Medicaid enrollees receive.”^37^ As the system moves to VBP, in which the report of the delivery of particular quanta of value is a condition of payment, one risk is that there will be a skewing toward cost-saving and not equity and social care gains, as the measurement of the latter two goals is more difficult than measuring cost savings.^38^

Early returns on the effects of VBP in Medicaid gives reason for concern. Researchers find little benefit to low-income and vulnerable populations, with some indication that, rather than improve equity and access, VBP methods have been harmful to vulnerable populations, even heightening inequities.^39^ It may be that the fault continues to be an overemphasis on cutting costs,^40^ which may unintentionally lead to underinvestment in the populations most in need.^41^

Two problems must be addressed if VBP methods is to be more than Managed Care 2.0, with no more success in improving equity and addressing HRSNs that previous ventures in payment innovation. First, it is important for CMS and the states implementing novel systems to be clear on what they mean by “value.” A vague laundry list of values, including cost savings, may frustrate attempts to drive providers to addressing HRSNs and equity — which may be difficult to measure and produce when compared to cost. CMS and states must be clear in communication with community providers and partners whether they’re hunting for Snarks or Boojums.^42^

Second, and the focus of the next part of this paper, is that CMS and states should focus not on general goals but instead on the *structures* of VBP systems. It may be more effective for states to evaluate providers and partners in VBP systems on their fidelity to structural design requirement, which can be evaluated readily, than on a list of general goals and standards, which can be difficult to administer.

The next part describes CC Hubs, which are a structural instantiation of the desire to connect vulnerable Medicaid beneficiaries with the social services that will advance equity goals and respond to their HRSNs.^43^ A concrete structure devoted to connecting patients to the social services responsive to their particular vulnerabilities can advance the equity and whole person goals of Medicaid, minimizing the chance that those goals will be lost in the shadow of sometimes-more-salient cost issues.

## II. CC Hubs

VBPs are a current iteration of desire to use the structure and signaling power of payment policy and implementation to drive improvements in health care delivery. Past efforts to engage in such signaling have fallen short in achieving the desired outcomes related to health equity and access to care for vulnerable populations in favor of cost containment.^44^ This is not a surprising result; cost is and will continue to be an important goal for all health payers, and measuring cost containment is easier than measuring other desired outcomes.^45^ There are, in addition, structural difficulties in driving health access and equity through payment mechanisms.

VBPs are more likely to succeed in driving the “softer” goals of equity and access if they include metrics for success that include measurable outcomes and processes indicative of attention to amelioration of HRSNs.^46^ There has been some work in creating, testing, and organizing the development of information that can identify successes and failures in adding HRSN-related value to service delivery.^47^ This paper addresses instead a structural issue: how can equity and access for vulnerable populations be centered in an aspect of the health care finance and delivery system such that it is less likely to be pushed to the side in VBP systems? An answer is CC Hubs.

### A. CC Hubs as intermediaries

CC Hubs concretize what is often a general focus on equity and access issues by locating these functions in a structure dedicated to the resolution of HRSNs. They are conceptually and structurally between:

- *medical entities*: hospitals and hospital systems, multispecialty physician practices, federally qualified health centers and other medical clinics, managed care entities and other health insurers, and self-funded health plans — all of which in a VBP world may have an incentive — even if not a mission — to seeing that patients’ HRSNs are addressed and remediated;^48^ and,
- *social service community-based organizations (CBOs)*: low-income housing providers and homelessness services agencies, food pantries and nutritional counseling organizations, reentry programs dedicated to the reintegration of prisoners returning to their communities, juvenile support organizations specializing in services for juveniles disconnected from their families — all of which have an interest and usually a mission to address specific HRSNs.^49^

CC Hubs bridge these two worlds. Health entities often have substantial organizational and technology capacities, tight health finance connections — health payers with large providers, and large providers with payers — and financing that through VBP conditions payment on metrics, including addressing HRSNs, but often poor connections to granular community-based services.^50^ To put a finer point on it, a multi-hospital system is often poorly connected with the many small CBOs best able to address HRSNs.^51^ CBOs on the other hand, are well-equipped to provide social services themselves or in collaboration with known community partners.^52^ What they lack is systems sophistication. They are not familiar with National Provider Identifier Standards (NPIs),^53^ often do not have the willingness or capacity to become an in-network providers in a Medicaid managed care plan,^54^ and do not wish to take on the arduous responsibility of billing Medicaid or Medicare.^55^

CC Hubs can address the shortfalls in both directions. For CBOs, CC Hubs

[O]rganize] and support[] a network of community-based organizations providing services to address health-related social needs. A CCH centralizes administrative functions and operational infrastructure, including, but not limited to, contracting with healthcare organizations, payment operations, management of referrals, service delivery fidelity and compliance, technology, information security, data collection, and reporting.

A CCH has trusted relationships with and understands the capacities of local community-based and healthcare organizations and fosters cross-sector collaborations that practice community governance with authentic local voices.^56^

For health providers and payers, CC Hubs:

[C]an ease the implementation burden that would otherwise come with ad hoc partnerships between CBOs and health care organizations. [CC Hubs] relieve health care organizations of finding, contracting with and managing multiple independent CBOs, who are both sufficiently grounded in their local communities and collectively span the often-large geographic regions under the health care organization’s charge. CBOs can partner with multiple health care organizations with more consistent policies and systems through just one contract with the [CC Hub].^57^

In addition, CC Hubs can serve as the “back office” for CBOs, providing personnel qualification information to health partners, receiving and transmitting payment for services, and gathering and transmitting data on “closed loops” — the successful provision of social services to patients.

CC Hubs provide the “connective tissue” to ensure good communications and proper coordination between medical and social providers. They take on administrative tasks, offering health care entities and CBOs a “single point of contact” into the cooperative relationship that can allow social care. This advances equity and access for vulnerable populations into a part of the VBP proposition.^58^

To be clear, CC Hubs are more than just a convenient component of an organizational chart. Instead, they can be a visible, practical method of institutionalizing a connection between health care and social care such that equity and access issues are unlikely to fall by the wayside. The success of CC Hubs may vary, but they can occupy a systemic location that can become *convenient* for health providers responsible by contract to address HRSNs and *mission sustaining* for CBOs with expertise and willingness to address HRSNs but too little visibility and institutional support.

### B. CC Hub example: New York State Social Determinant of Health Networks

Many state Medicaid reform efforts seek to address health outcomes by addressing access to HRSNs.^59^ These efforts often involve VBPs, which make possible addressing social factors “that may be upstream from the clinical encounter.”^60^ One challenge “to integrating social care into the health care setting is the administrative capacity of social services providers, which often are key to the delivery of social care.”^61^ New York State in a recently approved § 1115 Medicaid waiver,^62^ has taken on this challenge by empowering its version of CC Hubs.

The guiding conception of the current iteration of New York’s § 1115 Medicaid waiver was the revelation during and after the COVID-19 pandemic that health access and outcomes are unequal along class and racial lines in New York. To address these disparities will require attention to both health delivery and access to social services.^63^ Equity concerns revealed by the COVID-19 crisis are a significant driver of the waiver:

Black and Latino/Latinx populations accounted for higher levels of COVID-19 related hospitalizations and mortality than white populations … . The higher rates of COVID-19 cases, hospitalizations, and deaths among people of color — due to their higher prevalence of chronic illness, overrepresentation in frontline and essential jobs, increased likelihood of living in multi-family or multi-generational housing, and other factors — have illustrated how pervasive health inequities remain.^64^

Among the lessons drawn from the COVID-19 results was the importance of linking health care to social services, particularly for vulnerable populations. New York’s post-COVID lessons include the insight that attention to “sick care” and the restructuring of health and social services can “more successfully connect traditional health care services and SDH service systems, in order to take a more holistic approach to health care and address the health needs of the whole person.”^65^

The delivery of this social care will be coordinated through two sets of entities: Health Equity Regional Organizations (“HEROs”) and Social Determinant of Health Networks, since renamed Social Care Networks. (“SCNs”).^66^ HEROs are the planning organizations comprising “new or existing corporate entities” that can include local health departments and other regional health and social service organizations and will be responsible to create collaborations and “develop a range of VBP models or other targeted interventions suitable for the populations and needs of each region” of New York State.^67^

The SCNs are versions of CC Hubs. They “consist of a network of CBOs in each region of the State.”^68^ Their functions map onto the description of the intermediary roles of CC Hubs detailed above:

[Each of the nine SCNs] would be responsible for: 1) formally organizing CBOs to perform SDH interventions; 2) coordinating a regional referral network with multiple CBOs, health systems and other health care providers; 3) creating a single point of contracting for SDH arrangements; and 4) screening Medicaid enrollees for the key SDH social care issues and make appropriate referrals based on need. The SDHNs can also provide support to CBOs around adopting and utilizing technology, service delivery integration, creating and adapting workflows, and other business practices, including billing and payment. These SDHNs will coordinate and work with providers in MCO networks to more holistically serve Medicaid patients, particularly those from marginalized communities, effectively wrapping a social services provider network with existing MCO clinical provider networks.^69^

The SCNs, then, will provide “back office” support for CBOs, freeing them from the contracting tasks otherwise inherent in dealing with Medicaid MCOs and providers.^70^ They will organize referral networks, providing a “single point of contracting” for HRSN services, allowing Medicaid health providers easy access to providers of social services.^71^ In addition, the New York § 1115 waiver program provides direct funding for some HRSN services, including:

- Housing supports;
- Pre-tenancy services;
- Tenancy-sustaining services;
- Housing transition navigation services;
- Nutrition supports;
- Case management; and
- Transportation services.^72^

If New York’s § 1115 waiver program is fully implemented and allowed to develop,^73^ then, it will realize all the benefits of CC Hubs. In addition, it will include funding for some of the social services needed by Medicaid patients — but crucially, by directing the funding not to health providers (the “medical-industrial complex”), but to the CBOs that have been providing social services and will do so in conjunction with health services providers.^74^

New York’s waiver program, then, will be, as § 1115 waiver programs are meant to be, a true experimental pilot program testing the utility of CC Hubs on a wide scale over a significant period.

## III. Challenges to the use of hub models

### A. Medicalization of health-related social needs

The devotion of time and resources to the use of CC Hubs in general, and New York State’s Social Care Networks in particular, is rooted in the recognition that individuals’ health status is proportionately more a product of their social circumstances and health-related social needs than to their access to medical care;^75^ the percentages are variously estimated, but health services can be counted as only a ten to twenty percent causative factor in personal health.^76^ As discussed above, efforts to integrate access to social and medical care are seen by many as essential to addressing health equity and improving the health status of vulnerable populations.^77^

One important push-back to this movement to integrate medical and social care is to argue that this linkage threatens to “medicalize” concerns that have been regarded as properly located out of the health care delivery system and that properly lie outside the scope of medical care. “Medicalization” has been defined as “the process by which personal, behavioral, and social issues are increasingly viewed through a biomedical lens and ‘diagnosed and treated’ as individual pathologies and problem” rather than as problems for social actors separate from the health care system to address.^78^ The integration of the social and medical in one health finance and delivery system is criticized on the grounds of inappropriate medicalization on three bases.

First, the therapeutic relationship initiated by a patient’s appointment with a physician is more complex where the diagnostic process considers both medical and social issues.^79^ Physicians may be concerned that they are now expected to extend their expertise from the biomedical realm to the complex world of social services. While many medical schools have added a focus on social drivers of health,^80^ physicians are not, and do not, feel capable of becoming experts in connecting patients to needed social services.

Many fundamental determinants of health are far upstream of health care and are deeply rooted in the distribution of money and power … .

[P]hysician engagement with the social determinants is different than physicians’ core role: providing health care to those who need it. Through necessity, clinicians must focus on the immediate illness or health problem and are not well positioned to coordinate longer-term population-level health improvement strategies.^81^

The CC Hub model contains a structural response to that concern. The role of the physician and a social worker or care manager is limited to identifying the patient’s needs. It is the CC Hub that is responsible for accepting the referral for social care, referring it to a suitable CBO, and closing the loop with a report back to the referring health provider.^82^ The burden on physicians, while not inconsequential, seems consistent with their current practices of including social issues in their diagnoses.

Second, “medicalization” is raised in concerns that linking assessment and referral for social services within the medical diagnosis and treatment setting will pathologize social conditions that are not intrinsic to the patient but rather the result of structural faults in social systems. Under this critique, identifying housing instability or poverty as drivers of health that are swept into the ambit of the “medical industrial complex”^83^ can locate the problem (lack of housing) in the patient and not in society, with implications for both weakening the agency of the patient and increasing the power of the medical system at the expense of the patient’s liberty and autonomy.^84^

The concerns about medicalization in this sense should not be minimized. Patient care, however, can be improved by thoughtful integration of medical and social drivers of health. Webb and Matthew argue that the path to allowing good medicalization while avoiding bad medicalization is to carefully medicalize not the person but the risk factors, which they refer to as “causes of the causes” of poor health:

Medicalization is most appropriately a descriptive and operative framework to understand the comprehensive range of contributors that cause the need for medical care and intervention in the first place … . By their very nature, incorporation of the social determinants of health into clinical settings results in the medicalization of these socially created risk factors for poor health - that is, we use the clinical encounter as an opportune setting in which to identify and coordinate the address poverty-driven needs.^85^

Webb and Matthew see three benefits to this medicalization of risk factors: it permits more accurate assessment of the causes of poor health, combats the fragmentation of care by encouraging relationships between medical caregivers and social service providers, and creates a “business case for blending and braiding” funding for care.^86^

The use of CC Hubs fits within this framework of medicalization as a positive factor. Utilizing CC Hubs provides tangible pathways to address HRSN thereby allowing medical caregivers to open the diagnostic lens to social needs,^87^ encourage the interaction of providers of health and social services by engaging CC Hubs and their associated CBOs in care for the patient,^88^ and (as described below^89^) increase the visibility of information about the benefits of engaging social care in the health care setting, supporting arguments for fuller funding for integrated care.

Third, medicalization of social services is argued to divert focus from the community-based providers of social services in favor of medical providers, thereby shifting attention and funding from organizations long-committed to advocating for and improving access to services such as housing and nutritional services.^90^ Funding for housing services, for example, are grossly inadequate, with many families left to wait for many years for vouchers for subsidized housing.^91^

The CC Hub model does not incorporate funding for social services support into the health delivery system.^92^ Instead, it creates a mechanism to facilitate communication and collaboration between health providers and social service agencies in the community. An important aspect of the CC Hub model may, in fact, amplify the voice of social service providers seeking funding.

The CC Hub serves as an intermediary between the health system and the social service system. The intermediary function features important communication aspects. A service request originates in a health entity — a managed care plan, a hospital system, a physician practice, for example — in the form of an identification of a social service need of a patient of the health entity.^93^ The Hub evaluates the service request and transmits it to a suitable CBO with expertise and community connections evidencing an ability to fulfill the request.^94^ The CBO performs the work and “closes the loop” (that is, establishes not just a referral for services but the patient’s receipt of the social services requested) or reports an inability to close the loop.^95^ The CC Hub then transmits the result to the health entity, which in turn submits payment to the CC Hub which then submits payment to the CBO.^96^

This structure creates a golden opportunity for effective advocacy. The transactions provide information about whether resources are available to “close the loop” on a referral. Little tweaking would be required to include information about the reasons for failed referrals: lack of housing opportunities, restrictions on access to available services, or quality problems with available housing rendering it unsuitable for the patient referred. Such information can be gathered and transmitted to housing funders — local, state, and federal agencies and/or private philanthropies — to inform them of the gaps, inadequacies, and stumbling blocks in the local low-income housing market. The information flows both ways by virtue of the loop-closing mechanism of the CC Hub model, allowing nearly real-time reporting of the status and availability of the relevant services, and the steps necessary to fill gaps. The “medicalization,” then, can provide an opportunity for advocacy tied directly to active case studies.

Medicalization is an issue contested in its meaning and application in the integrated health setting. Care must be taken to avoid burdening overworked and ill-equipped health providers with the task of mastering social service referral opportunities, to avoid pathologizing social deficits external to the patient and traceable to social defaults, and to help rather than harm the ability of social service providers to flourish and obtain needed funding. The CC Hub model is structured in such a way as to tick these boxes, and to facilitate rather than impair the availability of social services in integrated settings.

### B. The shifting CMS stance on supporting projects addressing health related social needs

States have long used Medicaid funding, and in particular the flexibility provided through § 1115 waivers, to address HRSN.^97^ During the Biden Administration, CMS provided expansive guidance on the permissible uses of Medicaid for these purposes. In November 2023, a CMS Informational Bulletin encouraged states to use a number of Medicaid provisions, including § 1115 waivers, to connect beneficiaries to social services as necessary to meet those beneficiaries’ comprehensive health needs.^98^ The Informational Bulletin highlighted the social services fitting within CMS’s universe of social services appropriate for coverage by various Medicaid authorities.^99^ Those services are more fully described in an accompanying “framework of services” as including:

- Housing supports including security deposits, utilities activation fees, moving costs, first month’s rent, home remediation (including mold removal and carpet replacement) and refrigerators; and
- Nutritional counseling and education, assistance with health meal preparation, home delivered meals, and grocery provision.^100^

The provision of these non-medical services through a § 1115 waiver would, of course, need to be approved by CMS and comply with all waiver requirements^101^ including budget neutrality as to federal expenditures, necessity to serve the research goals of the waiver, and consistency with federal requirements for Medicaid spending.^102^

CMS followed up its 2023 guidance the following year, in which it further explained the importance of coverage of HRSN for the beneficiary populations:

Coverage of targeted HRSN services and supports is likely to assist in promoting the objectives of Medicaid because it is expected to help beneficiaries stay connected to coverage and access to needed health care. The housing and nutritional support services authorized in demonstrations are expected to stabilize the housing and nutritional situations of eligible Medicaid beneficiaries and thus increase the likelihood that they will keep receiving and benefiting from the Medicaid covered services to which they are entitled.^103^

These encouragements to states to use Medicaid tools, including § 1115 waivers, to serve the HRSNs of Medicaid recipients is consistent with a broad vision of Medicaid’s purpose to improve health status and not simply to address discrete diseases. The encouragement led to states’ decisions to seek and gain approval for § 1115 waivers consistent with the CMS vision:

These waivers authorize evidence-based housing and nutrition services for specific high-need populations. Several approvals build on prior 1115 waiver initiatives … . Approvals include coverage of rent/temporary housing and utilities for up to 6 months and meal support up to three meals per day (for up to 6 months), departing from longstanding prohibitions on payment of “room and board” in Medicaid … . [O]ther states have approved 1115 waivers with SDOH-related provisions that pre-date the new Biden administration HRSN framework. These waivers are generally narrower in scope (services and target populations) or pilot programs targeting specific regions.^104^

With the end of the Biden Administration and the second coming of the Trump administration, CMS has revised its guidance. On March 4, 2025, CMS published an Informational Bulletin titled, “Rescission of Guidance on Health-Related Social Needs.”^105^ Unsurprisingly, this guidance reverses the agency’s course and eliminates the effect of the 2023 and 2024 guidance documents discussed above.^106^ After recounting the previous guidance, this guidance announces:

To evaluate policy options consistent with Medicaid and CHIP program requirements and objectives, CMS is rescinding the November 2023 and December 2024 CIBs. CMS will consider states’ applications to cover these services and supports on a case-by-case basis … without reference to the November 2023 and December 2024 CIBs or the HRSN Framework.

CMS is committed to working with states to help identify strategies to support innovation and improvement in Medicaid and CHIP.^107^

In an additional guidance document dated April 10, 2025,^108^ CMS highlighted concerns with some states’ § 1115 waiver funding mechanisms.^109^ The letter singled out “designated state health program” (“DSHP”) and “designated state investment programs” (“DSIP”) for opprobrium.^110^ DSHP and DSIP funding have attracted attention in the past.

Medicaid’s shared funding system is premised, of course, on federal contributions pegged to the amount necessary to meet the needs of eligible beneficiaries in combination with state contributions pegged to the rate of low-income state residents.^111^ Simply stated, Medicaid funding intends to include financial matches from the federal and state governments, accounting for varying poverty levels among the states.^112^

In DSHP arrangements, states designate state-funded programs that “do not otherwise qualify for federal funding, … existed prior to 1115 waiver implementation, and often provide safety-net health care services for low-income or uninsured individuals (such as addiction recovery treatment or support for individuals with intellectual and developmental disabilities).”^113^ DSIPs are a similar mechanism, in which the state arrangements that benefit low-income or uninsured individuals address technology or infrastructure needs, and not patient care.^114^ Examples of DSHP and DSIP programs through which states counted their expenditures on state innovations as their Medicaid match for § 1115 waivers include: $20M for high-speed internet for rural health care providers in North Carolina;^115^ student loan repayment programs in California designed to incentivize health care providers to practice in high-needs regions;^116^ and a “diversity in medicine” program in New York^117^ that “offers pathways to medical school for students who have demonstrated their commitments to improving health disparities and/or practicing medicine in underserved communities.”^118^

Under previous practice, the funds states expended under DSHP and DSIP programs are counted as a state match for federal Medicaid funding for § 1115 waivers.^119^ As the April 10 letter noted, “States with approved DSHP authority [for funding their § 1115 waivers] include: Arizona, California, Hawaii, Massachusetts, New York, North Carolina, Oregon, and Washington.”^120^ The letter then drops the hammer on creative funding for § 1115 waivers:

CMS does not anticipate approving new state proposals for section 1115 demonstration expenditure authority for DSHP or DSIP or renewing existing section 1115 demonstration expenditure authority for DSHP or DSIP. As such, in addition to this letter, the Center for Medicaid and CHIP Services (“CMCS”) will conduct direct outreach to states with existing DSHP and DSIP authority to emphasize that the time-limited authority for DSHP or DSIP will not be extended beyond the currently approved demonstration period or, when current DSHP or DSIP authority concludes before the end of the demonstration’s approval period, the current end date for such authority.^121^

In the first few months of the second Trump administration, then, CMS has rescinded previous CMS guidance advocating broad use of the Medicaid program to provide for the HRSNs of Medicaid beneficiaries and has called into question one long-standing method used by states to create programs under § 1115 waiver authority to use innovative programs and structures to improve the lives of vulnerable Medicaid beneficiaries.^122^ It is unclear how far this disapproval will develop into a denial of state programs. As of this writing, no waivers have yet been rescinded, and no waiver applications for new programs or renewal of existing waivers have been denied. The guidance is worrying, but there is no indication of the extent to which care will be affected. The lasting the effects of CMS’s 2025 guidance are also unclear: will the position of CMS flip back in a future administration?

## IV. Conclusion

Medicaid has struggled to couple its funding of services with a mechanism for ensuring that its goals and intentions in providing that funding are embraced and acted upon by the health providers with which it partners. No aspect of this mismatch is clearer than the unmet goals of increasing health equity and addressing Medicaid beneficiaries’ health deficits traceable to health-related social needs. Medicaid managed care, a dominant mechanism used by states to drive policy goals through to providers, garners little traction in addressing equity and HRSNs.

The fault in the connection between Medicaid managed care and providers may lie less in the goals set for the program and more in the complexity and confusion that arise when providers’ compliance is measured against Medicaid’s general goals for health finance and delivery.

The CC Hub mechanism described in this paper embodies a clear focus on equity and social care. In addition, however, it concretizes the goals by locating the mechanisms for achieving them in a single-purpose entity that integrates with but stands apart from hospital systems and managed care entities on the one hand, and the community-based organizations necessary to address equity and social service goals on the other. New York State’s § 1115 waiver program systemically created and supports these entities throughout the state and will test the model — should federal support continue to be forthcoming — perhaps allowing Medicaid to achieve long-sought goals of advancing the health status of its most vulnerable beneficiaries.

## Footnotes

1
*See* Elizabeth Hinton & Amaya Diana, *Medicaid Authorities and Options to Address Social Determinants of Health*, KFF (Jan. 29, 2024), https://www.kff.org/medicaid/medicaid-authorities-and-options-to-address-social-determinants-of-health-sdoh/ [https://perma.cc/MU96-4QCV] (discussing the role of Medicaid in providing coverage for those with greater social needs).

2 Robin Rudowitz et al., *Medicaid 101*, KFF (May 28, 2024), https://www.kff.org/medicaid/health-policy-101-medicaid?entry=table-of-contents-how-has-medicaid-evolved-over-time [https://perma.cc/DK6K-Q499].

3
*See* Elizabeth Hinton & Amaya Diana, *Medicaid Authorities and Options to Address Social Determinants of Health*, KFF (Jan. 29, 2024), https://www.kff.org/medicaid/medicaid-authorities-and-options-to-address-social-determinants-of-health-sdoh/#:~:text=How%20Can%20Medicaid%20be%20Used,to%20add%20non%2Dclinical%20benefits [https://perma.cc/5EQK-EPVS]; MACPAC, Financing Strategies to Address the Social Determinants of Health in Medicaid 2 (2022), https://www.macpac.gov/wp-content/uploads/2022/05/SDOH-Issue-Brief_May-2022.pdf [https://perma.cc/FM8B-5BUY].

4
*See* Joshua Breslau et al., Approaches to Coordination of Health and Social Care: An Overview of Backbone Organizations and Key Takeaways from Case Studies of Community Care Hubs iii, 3-4 (2023), https://www.ncbi.nlm.nih.gov/books/NBK604800/ [https://perma.cc/6NWX-6RTJ] (discussing some advantages of CC Hubs including improved care coordination and avoiding the need for individual negotiation).

5

Corinne Lewis et al., *Value Based Care: What It Is and Why It’s Needed (explainer)*, Commonwealth Fund (Feb. 7, 2023), 10.26099/fw31-3463 .

6
*See* Nat’l Acads. of Scis., Eng’g, & Med., Implementing High-Quality Primary Care 281-320 (Sarah K. Robinson, Marc Meisnere, Robert L. Phillips, Jr. & Linda McCauley eds. 2021) (discussing various payment structures).

7 For terms and conditions for hospital participation in Medicare and Medicaid in the U.S., see, for example, Conditions of Participation for Hospitals 42 C.F.R. § 482.1 (2024). *See also Quality, Safety & Oversight — Certification & Compliance*, Ctrs. for Medicare & Medicaid Servs. (Aug. 9, 2025), https://www.cms.gov/medicare/health-safety-standards/certification-compliance [https://perma.cc/XZ7R-NWY2].

8
*See* Roosa Tikkanen et al., *International Health Care System Profiles: France*, Commonwealth Fund (June 5, 2020), https://www.commonwealthfund.org/sites/default/files/2020-12/2020_IntlOverview_FRANCE.pdf [https://perma.cc/MR57-PDV7]. In France, for example, the services covered for all in the nation are determined by the Ministry of Social Affairs, Health, and Women’s Rights, and they declare priorities through mandated coverage for certain procedures, including some preventive care. *Id.*

9 AMA Principles of Medical Ethics (Am. Med. Ass’n 2001) (discussing a physician’s right, except in certain circumstances, to choose who they serve).

10 Olga Yakusheva et al., *Value-based Payment Promotes Better Patient Care, Incentivizes Health Care Delivery Organizations to Improve Outcomes and Lower Costs, and Can Empower Nurses*, 70 Nursing Outlook 215, 215-18 (2022).

11
*See infra* text accompanying notes 33-35. Health related social needs (HRSNs) are derived from an individual’s social determinants of health (SDOH). *Coverage of Services and Supports to Address Health-Related Social Needs in Medicaid and the Children’s Health Insurance Program*, Ctrs. for Medicare & Medicaid Servs. (Nov. 16, 2023), https://www.medicaid.gov/federal-policy-guidance/downloads/cib11162023.pdf [https://perma.cc/EQZ7-JFJF]. The Centers for Medicare and Medicaid Services (CMS), Center for Medicaid and CHIP Services (CMCS) has explained that SDOH refers to an individual’s determinants of health deriving from “broad environmental conditions,” while HRSNs “are specific to an individual and when unmet, these individual-level adverse social conditions contribute to poor health outcomes.” *Id.* CMS has further explained the difference between SDOH and HRSN in the following terms: Examples of SDOH include economic stability, access to quality education and health care, and the neighborhood and built environment. The specific factors that impact individuals directly are called “health-related social needs” (HRSN). Examples of HRSN include lack of stable or affordable housing and utilities, financial strain, lack of access to healthy food, personal safety, and lack of access to transportation. SDOH and HRSN can coincide and overlap.
*Social Drivers of Health and Health-Related Social Needs: Defining Key Terms*, Ctrs. for Medicare & Medicaid Servs. (Feb. 27, 2025), https://www.cms.gov/priorities/innovation/key-concepts/social-drivers-health-and-health-related-social-needs [https://perma.cc/KS4Y-6S4R].

12
*See* Diogo L. L. Leao et al., *The Impact of Value-Based Payment Models for Networks of Care and Transmural Care: A Systematic Literature Review*, 21 Applied Health Econ. & Pol’y 441, 441-46 (Feb. 1, 2023) (discussing the risks of VBPs and the issues targeted by Medicaid programs, noting that without proper incentives, these programs can fall short of their goal).

13
*See* Andre Chappel et al., *Improving Health And Well-Being Through Community Care Hubs*, Health Affs.: Forefront (Nov. 29, 2022), 10.1377/forefront.20221123.577877 (discussing the role of community care hubs); *see also infra* text accompanying note 48-50.

14 U.S. Dep’t Health & Hum. Servs., Community Care Hubs: A Promising Model for Health and Social Care Coordination ii-iii (2023), https://www.ncbi.nlm.nih.gov/books/NBK604800/ [https://perma.cc/6NWX-6RTJ]; Breslau, *supra* note 4, at 22.

15
*See* Breslau, *supra* note 4, at 1, 3-4 (discussing the relationship between CC Hubs and CBOs and the potential to improve “a broad variety of health outcomes” for patients with HRSNs).

16
*See id.* at 15-30 (discussing the advantage of community focused organizations as allowing community members to provide feedback in contrast to payers and systems that are “distrusted and seen as large and distant organizations”).

17
*See id.* (discussing networks created by CC Hubs and CBOs that allow for these relationships).

18
*See generally* William M. Sage & Jennifer E. Laurin, *If You Would Not Criminalize Poverty Do Not Medicalize It*, 46 J.L., Med. & Ethics 573 (2018) (criticizing the medicalization of property); *see* infra text accompanying notes 78-91.

19
*See generally* Sage & Laurin, *supra* note 19; *see also* Paula M. Lantz et al., *The Perils of Medicalization for Population Health and Health Equity*, 101 Milbank Q. 61, 63 (2023) (suggesting that medicalization fuels an overemphasis on health services as the primary avenue for promoting health, presenting a challenge to improving population health).

20
*See* Ctrs. for Medicare & Medicaid Servs., Opinion Letter on Use of Section 1115 Waivers to Obtain Federal Funds for DSHP or DSIP Funding (Apr. 10, 2025), https://www.medicaid.gov/resources-for-states/downloads/dshp-dsip.pdf [https://perma.cc/TM83-BS29] (addressing CMS concerns of providing federal funding for DSHP and DSIP); *infra* text accompanying notes 105-110.

21
*See* Irene Papanicolas et al., *The Relationship Between Health Spending and Social Spending in High-Income Countries: How Does The US Compare?*, 38 Health Affs. 1567, 1567 (2019) (discussing how U.S. spends nearly twice the average of GDP on its health care system yet with relatively poor health outcomes while having below average social spending). For further discussion, see generally Elizabeth H. Bradley et al., The American Health Care Paradox: Why Spending More is Getting Us Less (2013) (exploring the paradox of higher spending versus equal or poorer outcomes in the US compared to other countries).

22
*See* Nicole Huberfeld, *Is Medicare for All the Answer? Examining the Health Reform Gestalt as the ACA Turns 10*, 20 Hou. J. Health L. & Pol’y 69, 85-93 (2020) (explaining the structure of “Medicare for All”); *see also* Lindsay F. Wiley, *Privatized Public Health Insurance and the Goals of Progressive Health Reform*, 54 U.C. Davis L. Rev. 2149, 2179-85 (2021) (discussing place of private insurance in a reformed public system).

23
*See* Erin E. Fuse Brown et al., *Social Solidarity in Health Care*, *American Style*, 48 J.L., Med. & Ethics 411, 417-19 (2020) (advocating for solidarity as an organizing principle for health reform); *see also* Gabriel Scheffler, *Equality and Sufficiency in Health Care Reform*, 81 Md. L. Rev. 144, 162-63 (2021) (asking whether goal of health reform should be “sufficient” access or “equal” access).

24 Gwendolyn Roberts Majette, *From Concierge Medicine to Patient-Centered Medical Homes: International Lessons & the Search for a Better Way to Deliver Primary Care in the U.S.*, 35 Am. J.L. & Med. 585, 586 (2009); *see also* Sallie Thieme Sanford, *Designing Model Homes for the Changing Medical Neighborhood: A Multi-Payer Pilot Offers Lessons for ACO and PCMH Construction*, 42 Seton Hall L. Rev. 1519, 1520-23 (2012) (discussing how improvements in medical care infrastructure and structures of hospitals improves medical outcomes).

25
*See* Douglas A. Conrad, *The Theory of Value-Based Payment Incentives and Their Application to Health Care*, 50 Health Servs. Rsch. 2057, 2060 (2016) (discussing a version of agency theory in which the payer seeks to incentivize the agent to use funding to achieve payer’s goals).

26 John V. Jacobi, *Payment Theory and the Last Mile Problem*, 48 J.L., Med. & Ethics 474, 476 (2020).

27
*See* Blake N. Schultz et al., *Targeting Health-Related Social Risks in the Clinical Setting: New Policy Momentum and Practice Considerations*, 51 J.L., Med. & Ethics 777, 778 (2023) (“There are numerous, interacting social and environmental conditions associated with poor health, including housing instability and food insecurity” and addressing HRSNs can improve health status and also can “present a substantial opportunity to advance health equity.”).

28 Michael Sparer, Robert Wood Johnson Found., Medicaid Managed care: Costs, Access, and Quality of Care 15-16 (2012), https://www.cancercarediff.org/wp-content/uploads/2020/12/managed-care-rwjf.pdf [https://perma.cc/VMP8-XY3F].

29
*See id.* at 18-19 (discussing mixed findings regarding impact on quality of care).

30
*Managed Care’s Effect on Outcomes*, MACPAC (Sep. 12, 2023) (alteration in original), https://www.macpac.gov/subtopic/managed-cares-effect-on-outcomes/ [https://perma.cc/B26L-E4VH].

31
*See id.* This paper does not address other structural attempts to improve access to social care and health equity in Medicaid. One method, situated temporally between Medicaid Managed Care and VBP efforts is the Medicaid Accountable Care Organization (“ACOs”). *See* Elizabeth Tobin-Tyler & Benjamin Ahmad, Milbank Mem’l Fund, Marrying Value-Based Payment and the Social Determinants of Health through Medicaid ACOs 3, 10-17 (2020), https://www.milbank.org/wp-content/uploads/2020/05/Medicaid-AC0s-and-SD0H.ver5_.pdf [https://perma.cc/5W2A-N5P8]. These organizations, often the creatures of § 1115 waivers, are quite heterogeneous in structure, and the study of their efficacy in serving social care and equity goals is ongoing, although some states’ experience is promising. *See id*; Meredith B. Rosenthal et al., *Realizing the Potential of Accountable Care in Medicaid*, Commonwealth Fund (Apr. 12, 2023), 10.26099/jx0w-4q30 .

32 Jessica Mantel, *Health Care Finance Law’s Relational Bias*, *in* Health Law as Private Law 229, 230 (I. Glenn Cohen et al. eds., 2025).

33
*Value-Based Payment*, MACPAC (Mar. 20, 2020), https://www.macpac.gov/subtopic/value-based-purchasing/ [https://perma.cc/B6VN-P24U].

34 Sylvia Mathews Burwell, *Progress Towards Achieving Better Care, Smarter Spending, Healthier People*, U.S. Dep’t of Health & Hum. Servs.: Blog (Jan. 26, 2015), https://wayback.archive-it.org/8315/20170118233619/https://www.hhs.gov/blog/2015/01/26/progress-towards-better-care-smarter-spending-healthier-people.html.

35
*See id.*

36 Sparer, *supra* note 28, at 15, 19, 22.

37
*Id.* at 22.

38
*See* Conrad, *supra* note 25, at 2061-62 (describing the importance in a VBP system of accurate measurement of the value markers to avoid a shift to purely financial incentives).

39 Joshua Liao et al., *A National Goal to Advance Health Equity Through Value-Based Payment*, 325 [J]AMA 2439, 2439 (2021).

40
*See* Suhas Gondi et al., *“REACHing” for Equity — Moving from Regressive toward Progressive Value-Based Payment*, 387 New Eng. J. Med. 97, 99 (2022).

41
*Id.* at 97.

42
Just the place for a Snark! I have said it twice: That alone should encourage the crew. Just the place for a Snark! I have said it thrice: What I tell you three times is true. … . In the midst of the word he was trying to say, In the midst of his laughter and glee, He had softly and suddenly vanished away— For the Snark was a Boojum, you see. Lewis Caroll, The Hunting of the Snark: An Agony in Eight Fits 7, 38 (The Floating Press 2018) (1876).

43
*See* P’ship to Align Social Care, Community Care Hubs: Making Social Care Happen: Background, Evolution, and Value Proposition of Working with a Local CBO Network Led by a Community Care Hub 1, 2 (2022), https://www.partnership2asc.org/wp-content/uploads/2022/12/CCH-Primer-Final.pdf [https://perma.cc/8SLU-N8HK].

44
*See* Liao, *supra* note 39, at 2439 (discussing the failure of various payment programs to address inequity).

45
*See* Larry Levitt et al., *Quality of Care: Assessing the Performance of the U.S. Health System*, KFF (Nov. 30, 2014), https://www.healthsystemtracker.org/brief/assessing-the-cost-and-performance-of-the-u-s-health-system/#:~:text=Health%20outcomes%2C%20or%20what%20we,on%20any%20amount%20of%20improvement [https://perma.cc/RS5R-HZMK].

46 See Diana Crumley, *Medicaid and Health-Related Social Needs: Four Insights over Four Years*, Ctrs. for Health Care Strategies (Sep. 10, 2021), https://www.chcs.org/medicaid-and-health-related-social-needs-four-insights-over-four-years/ [https://perma.cc/SRS7-LSNZ].

47
*See, e.g.*, Hannah L. Crook et al., Milbank Mem’l Fund, How Are Payment Reforms Addressing Social Determinants of Health? Policy Implications and Next Steps 6-8 (2021), https://healthpolicy.duke.edu/sites/default/files/2021-02/How%20Are%20Payment%20Reforms%20Addressing%20Social%20Determinants%20of%20Health.pdf [https://perma.cc/TQJ2-66YC]. Vermont’s all-payer ACO model used its all-payer claims database and the Vermont Health Information Exchange as well as a dashboard tool showing medical and nonmedical indicators collected during encounters. Colorado recently proposed a two-phase social-health information exchange. In phase I of the rollout of Colorado’s exchange, organizations developed standardized screening protocols, built a statewide community resource directory, and made the directory publicly available. Phase II consists of linking existing information systems, developing referral and feedback loops, and storing SDoH screenings in a patient data management system. Similar initiatives are also underway in states such as Indiana and Nebraska. North Carolina has launched NCCARE360 to provide a common channel for making referrals for social and community-based services, which facilitates two-way communication between health care and social services.
*Id.* at 6 (footnotes omitted).

48
*See* Breslau et al., *supra* note 4, at iii, vii.

49
*See id.* at vii; Diana Crumley et al., Ctr. for Health Care Strategies, Incorporating Community-Based Organizations in Medicaid Efforts to Address Health-Related Social Needs: Key State Considerations 9-10 (2023), https://www.chcs.org/media/Incorporating-Community-Based-Organizations-in-Medicaid-Efforts-to-Address-Health-Related-Social-Needs_050523-1.pdf [https://perma.cc/V36H-RC4Q].

50
*See* Kerry Griffin et al., Greater N.Y. Hosp. Ass’n, N.Y. Acad. Med., Partnerships Between New York City Health Care Institutions and Community-Based Organizations: A Qualitative Study on Processes, Outcomes, Facilitators, and Barriers to Effective Collaboration 5, 9 (2018), https://collections.nlm.nih.gov/master/borndig/101735783/hco_cbo_partnerships_digital.pdf [https://perma.cc/V8E7-7CPX] (addressing the ability of CBOs to improve the community coordination of health care organizations); *see also* Nat’l Comm. for Quality Assurance, Co-Developing Cross-Sector Partnerships to Address Health-Related Social Needs: A Toolkit for Health Care Organizations Collaborating With Community-Based Organizations 9-10 (2024), https://wpcdn.ncqa.org/www-prod/wp-content/uploads/NCQA-HCO-Toolkit.pdf#:~:text=PRIORITIZE%20TRANSPARENCY%20BEFORE%20FORMALIZING%20AGREEMENTS,CBOs%20might%20have%20capacity%20challenges [https://perma.cc/J3BW-HRGQ] (discussing partnerships between health care organizations and CBOs).

51
*See* Nat’l Comm. for Quality Assurance, *supra* note 51, at 9 (noting that where HCO business units overlap on initiatives, CBOs can be confused about which to engage).

52
*See id.* at 17.

53
*National Provider Identifier Standard (NPI)*, Ctrs. for Medicare & Medicaid Servs. (Sep. 10, 2024, at 18:21 ET), https://www.cms.gov/regulations-and-guidance/administrative-simplification/nationalprovidentstand [https://perma.cc/UA2S-RR8B]. A National Provider Identification number is a unique identifier for persons or entities participating in Medicare, Medicaid, and/or Children’s Health Insurance Program systems. *Id.*

54
*See* P’ship for Healthy Outcomes, Using Medicaid Levers to Support Health Care Partnerships with Community-Based Organizations 3 (2017), https://www.chcs.org/media/CBO-HCO-Partnership-Medicaid-Fact-Sheet_102417_chcs.pdf [https://perma.cc/KM5W-9SG2] (identifying challenges faced by partnerships between CBOs and health care organizations, including collection of partnership impact metrics and patient data-sharing); *see also* Agonafer et al., *Community-Based Organizations’ Perspectives on Improving Health and Social Service Integration*, 21 BMC Pub. Health, Mar. 6, 2021, at 1, 6, 8 (describing CBOs’ “wariness” of implementation of a § 1115 in California as stemming from difficulties in communication and lack of understanding of the Medicaid implementation protocols).

55
*See Unite Us and Nomi Health Partner to Accelerate and Streamline Payments for Community-Based Organizations Serving Medicaid Managed Care Organizations*, Bus. Wire (July 23, 2023), https://www.businesswire.com/news/home/20250723566547/en/Unite-Us-and-Nomi-Health-Partner-to-Accelerate-and-Streamline-Payments-for-Community-Based-Organizations-Serving-Medicaid-Managed-Care-Organizations [https://perma.cc/Y9ZX-KPK5] (announcing partnership between technology and financial services platform to address “slow complex payment processes” for CBOs); *see also* P’ship for Healthy Outcomes, *supra* note 54, at 3-4 (describing how Medicaid and managed care organizations can address CBO payment challenges through implementation of value-based purchasing contracts and authorization of social service coverage).

56 P’ship to Align Social Care, *supra* note 43, at 2.

57 Dutton et al., Manatt Health, Working With Community Care Hubs to Address Social Drivers of Health 8 (2022), https://assets-us-01.kc-usercontent.com/9fd8e81d-74db-00ef-d0b1-5d17c12fdda9/1f285a63-b073-4ddf-a2b0-725853bd3dfa/Manatt-CCH-Medicaid-Playbook_Final-11-17-22.pdf [https://perma.cc/T9SY-NT7P].

58 Chappel et al., *supra* note 13; Breslau et al., *supra* note 4, at vii.

59
*See* Karen B. DeSalvo & Michael O. Leavitt, *For an Option to Address Social Determinants of Health, Look to Medicaid*, Health Affs.:Forefront (July 8, 2019), https://www.healthaffairs.org/content/forefront/option-address-social-determinants-health-look-medicaid [https://perma.cc/BJ6M-KUHY] (outlining various strategies by state Medicaid plans to incorporate SDOH-related activities).

60 Nat’l Acad. of Scis., Eng’g, & Med., Integrating Social Care into the Delivery of Health Care 1 (2019).

61
*Id.* at 127.

62
*See infra* text accompanying notes 98-99; 42 U.S.C. § 1315(a). The Secretary of the Department of Health and Human Services may waive standard provisions of the Medicaid statute to allow states to engage in an “experimental, pilot, or demonstration project which, in the judgment of the Secretary, is likely to assist in promoting the objectives of” Medicaid. § 1315(a).

63
*See* Off. of Health Ins. Programs, N.Y. State Dep’t of Health, 1115 Waiver Demonstration: Conceptual Framework 3 (2021), https://www.health.ny.gov/health_care/medicaid/redesign/2021/docs/2021-08_1115_waiver_concept_paper.pdf [https://perma.cc/WM4V-XCGK] [hereinafter Conceptual Framework]. The § 1115 waiver was approved by letter in 2024, with waiver authority to run until March 31, 2027, unless the waiver is renewed. Amir Bassari, Ctr. for Medicaid & CHIP Servs. Approval of Request to Amend Section 1115(a) Waiver (Jan. 9, 2024), https://www.medicaid.gov/sites/default/files/2024-01/ny-medicaid-rdsgn-team-appvl-01092024.pdf [https://perma.cc/UJH5-GDAQ].

64 Conceptual Framework, *supra* note 64, at 5.

65
*Id.* at 7-8.

66
*Id.* at 8-9, 13; *see Social Care Networks (SCN)*, N.Y. State Dep’t of Health (Aug. 2025), https://www.health.ny.gov/health_care/medicaid/redesign/sdh/scn/ [https://perma.cc/L7GA-SWCE] (reflecting change in nomenclature from Social Determinant of Health Networks to Social Care Networks).

67 Conceptual Framework, *supra* note 64, at 9-10.

68
*Id.* at 13-14.

69 Conceptual Framework, *supra* note 64, at 14; *see Social Care Networks (SCN)*, *supra* note 67 (noting establishment of nine SCN regional entities).

70
*See* N.Y. State Dep’t of Health, Social Care Network: Program, Billing, and Data Governance Operations Manual 15-16, 234 (2025), https://www.health.ny.gov/health_care/medicaid/redesign/sdh/scn/docs/operations_manual.pdf [https://perma.cc/7W4V-JB5T].

71 Chappel et al., *supra* note 13 (describing the support SCNs can provide for CBO infrastructure based on Department of Health and Human Services’ strategy).

72 Alice Lam & Olga Deshchenko, *New York State’s Approved Health Equity 1115 Waiver Amendment: Summary of Key Provisions*, Manatt (Feb. 20, 2024), https://www.manatt.com/insights/newsletters/health-highlights/new-york-states-approved-health-equity-1115-waive [https://perma.cc/YPL6-XXVH].

73
*See infra* text accompanying notes 106-11(describing recent criticism from the Centers for Medicaid and CHIP Services of some §1115 waiver programs); Ctrs. for Medicare & Medicaid Servs., *supra* note 21 (criticizing some states’ § 1115 waiver programs, and calling out New York’s in particular, for their methods of funding the waiver programs).

74
*See* Eric Y. Frimpong et al., *Impact of the 1115 Behavioral Health Medicaid Waiver on Adult Medicaid Beneficiaries in New York State*, 54 Health Servs. Rsch. 677, 678 (2021) (noting the funding benefits of the 1115 waiver).

75
*See supra* note 11 and accompanying text.

76
*See* Sanne Magnan, *Social Determinants of Health 101 for Health Care*, Nat’l Acad. Med. (Oct. 9, 2017), https://nam.edu/wp-content/uploads/2017/10/Social-Determinants-of-Health-101.pdf [https://perma.cc/8D5A-7SUP].

77
*See* discussion *supra* Section I.B.

78 Lantz et al., *supra* note 19, at 63.

79
*See* Erin P. Balogh et al., Improving Diagnosis In Health Care 31-32, 35-36, 52 (Comm. on Diagnostic Error in Health Care et al. eds., 2015) (describing the complex nature of a diagnostic process that takes social issues into account).

80
*See* Loel S. Solomon et al., *What Are We Teaching Medical Students and Physician Learners About the Social Determinants of Health? A Scoping Review*, 100 Acad. Med. 103, 109-11 (2025) (“Leaders in medical education are engaging in a robust dialogue about the appropriate size and scope of curricula about the SDOH in physician training.”) (footnotes omitted); Joy Lewis et al., *Addressing the Social Determinants of Health in Undergraduate Medical Education Curricula: A Survey Report*, 11 Advances Med. Ed. & Prac. 369, 369, 373 (2020) (reporting that medical schools “are successfully integrating SDH-related content into UME curricula”).

81 Nason Maani & Sandro Galea, *The Role of Physicians in Addressing Social Determinants of Health*, 323 [J]AMA 1551, 1551 (2020).

82
*See infra* text accompanying notes 94-97.

83 Paul Starr, The Social Transformation of American Medicine 428-29 (1982), *quoted in* B. Cameron Webb & Dayna Bowen Matthew, *Housing: A Case for the Medicalization of Poverty*, 46 J.L., Med. & Ethics 588, 589 (2018).

84
*See* Sage & Laurin, *supra* note 18, at 574; Webb & Matthew, *supra* note 83, at 589 (ascribing to Michel Foucault the view that “medicalization operated to create an abuse power structure of control of physicians over patients”).

85 Webb & Matthew, *supra* note 83, at 591.

86
*Id.*

87 Crumley et al., *supra* note 49, at 7.

88
*See* Breslau et al., *supra* note 4, at 33-36 (describing the role of CC Hubs in coordinating health and social care services).

89
*See infra* Section III.B.

90
[T]he medicalization of the social determinants of health may also absolve policy makers in nonhealth sectors, such as justice, transportation, housing, employment, environment, benefits, education, sanitation, and a host of other vital contributors to health, from any responsibility in the generation of health, despite the widely accepted acknowledgment of these very same factors as crucial in determining health. Maani & Galea, *supra* note 81, at 1551; *see also* Sage & Lauren, *supra* note 19, at 575 (discussing the skewing of resources to alleviate poverty toward medical uses); Mitchell H. Katz, *Homelessness—Challenges and Progress*, 318 [J]AMA 2293, 2294 (2017) (“Having Medicaid pay for supportive housing in cases of persons with chronic medical or psychiatric illness would help to solve the funding problem for many homeless persons.”) (citation omitted).

91
*See* Sonya Acosta & Erik Gartland, Families Wait Years for Housing Vouchers Due to Inadequate Funding 1 (2021).

92
*See* Breslau et al., *supra* note 4, at 34-36 (describing that while CBOs provide care coordination, funding remains a barrier to their function).

93 Chappel et al., *supra* note 13.

94
*See id.*; Breslau et al., *supra* note 4, at 34-36.

95
*See, e.g.*, Breslau et al., *supra* note 4, at 66 (discussing one CC Hub’s reporting of whether referrals went through resulting in a “closed loop”).

96
*See id.* at 25 (discussing the use of CC Hubs to coordinate payment between CBOs and payers); Chappel et al., *supra* note 13 (modeling CC Hubs role in coordinating funding).

97
*See, e.g.*, Alexander P. Phillips et al., *Medicaid Section 1115 Waivers: From Work Requirements to Social Determinants of Health*, Health Affs.: Forefront (Apr. 20, 2023), 10.1377/forefront.20230418.28499 ; Ann G. Taylor & James E. Shmerling, *What is Medicaid and How Can/Should it be Used to Improve the Health of Our Nation’s Children*, 22 Quinnipiac Health L.J. 405, 431-34 (2019); HH Jonah Kushner & K. John McConnell, *Addressing Social Determinants of Health through Medicaid: Lessons from Oregon*, 44 J. Health Pol., Pol’y & L. 919, 919-21 (2019).

98
*See* Ctrs. for Medicare & Medicaid Servs., Coverage of Health-Related Social Needs (HRSN) Services in Medicaid and the Children’s Health Insurance Program (CHIP) 1 (2023), https://www.medicaid.gov/health-related-social-needs/downloads/hrsn-coverage-table.pdf [https://perma.cc/5BNT-99VV].

99
*Id.* at 2-7.

100
*Id.*

101
*See* 42 U.S.C. § 1315; 42 C.F.R. § 431.400 (2024).

102
*See* 42 U.S.C. § 1315; 42 C.F.R. § 431.400; Catherine McKee & Jane Perkins, Nat’l. Health L. Program, Primer: State Plan Amendments v. Section 1115 Waivers 4-5 (2021), https://healthlaw.org/wp-content/uploads/2021/06/Primer-on-SPA-v.-1115-FINAL-1.pdf [https://perma.cc/WR4B-5PCE].

103 Ctrs. for Medicare & Medicaid Servs., Coverage of Services and Supports to Address Health-Related Social Needs in Medicaid and the Children’s Health Insurance Program 2 (2024), https://www.medicaid.gov/federal-policy-guidance/downloads/cib12102024.pdf [https://perma.cc/4RCS-CA4B].

104 Hinton & Diana, *supra* note 3.

105
*See generally* Ctr. for Medicaid & CHIP Servs., Rescission of Guidance on Health-Related Social Needs (2025), https://www.hhs.gov/guidance/sites/default/files/hhs-guidance-documents/AID/cib03042025.pdf [https://perma.cc/QWW7-AN97].

106
*Id.*

107
*Id.*

108 Ctrs. for Medicare & Medicaid Servs., *supra* note 20.

109
*Id.*

110
*Id.*

111
*See* 42 U.S.C. § 1396d(b).

112
*See Medicaid 101*: *Financing*, MACPAC, https://www.macpac.gov/medicaid-101/financing/ [https://perma.cc/D5EH-GTHW] (last visited Sep. 5, 2025) (“The federal share for most Medicaid service costs is determined by the federal medical assistance percentage (FMAP), which is based on a formula that provides higher reimbursement to states with lower per capita incomes relative to the national average (and vice versa). The formula is intended to reflect states’ differing abilities to fund Medicaid from their own revenues.”); Elizabeth Williams, et al., *Medicaid Financing: The Basics*, KFF (Jan. 29, 2025), https://www.kff.org/medicaid/issue-brief/medicaid-financing-the-basics/ [https://perma.cc/GZ88-QUZL] (“Medicaid is administered by states within broad federal rules and jointly funded by states and the federal government through a federal matching program with no cap.”).

113 Amaya Diana, Elizabeth Hilton & Robin Rudowitz, *Section 1115 Waiver Watch: Early Signs Point to New Directions Under Trump Administration*, KFF (May 2, 2025), https://www.kff.org/medicaid/issue-brief/section-1115-waiver-watch-early-signs-point-to-new-directions-under-trump-administration/ [https://perma.cc/CRD2-DXG2].

114
*See* Ctrs. for Medicare & Medicaid Servs., *supra* note 20 (“[I]n 2021, CMS approved authority for expenditures, referred to as DSIP, for designated programs that provide or support the provision of health-related services that were otherwise state-funded and are not eligible for Medicaid funding in one state’s section 1115 demonstration.”).

115 Ctrs. for Medicare & Medicaid Servs., *CMS Refocuses on its Core Mission and Preserving the State-Federal Medicaid Partnership* (Apr. 10, 2025), https://www.cms.gov/newsroom/press-releases/cms-refocuses-its-core-mission-and-preserving-state-federal-medicaid-partnership [https://perma.cc/8AA3-YVU9] [hereinafter CMS Press Release].

116
*See California State Loan Repayment Plan (SLRP)*, Cal. Dep’t of Health Care Access & Info., https://hcai.ca.gov/workforce/financial-assistance/loan-repayment/slrp/ [https://perma.cc/Y27F-VVN9] (last visited Sep. 5, 2025).

117 CMS Press Release, *supra* note 115.

118 N.Y. State Dep’t of Health, *New York State Department of Health Announces Additional Funding to Continue Efforts to Improve and Expand Physician Diversity* (July 17, 2024), https://www.health.ny.gov/press/releases/2024/2024-07-17_doctor_tuition.htm [https://perma.cc/5EMY-4DTA].

119 Ctrs. for Medicare & Medicaid Servs., *supra* note 20.

120
*Id.*

121
*Id.*

122
*See* CMS Press Release, *supra* note 115.

